# The Prevalence and Cause of Dental Caries

**Published:** 1908-07-18

**Authors:** A. Louis George


### DENTAL DEPARTMENT.

### THE PREVALENCE AND CAUSE OF DENTAL CARIES.

By A. LOUIS GEORGE, L.D.S. ENG.

DENTAL caries is the most prevalent of all the diseases from which civilised man suffers: an extensive examination of ancient skulls reveals the fact that while caries was not unknown to primitive man, it did not exist in anything approaching the extent seen to-day.

An examination of the teeth of English and Scotch girls and boys, carried out some time ago by the British Dental Association, showed that in 10,500 children averaging twelve years of age there were no less than 37,000 decayed teeth, and 86 per cent. had caries in one form or another; these were children in Poor-law schools. No better result followed the examination of children of well-to-do parents: among 560 boys of similar age at one of the large public schools 701 of the permanent teeth had been lost and 3,521 were carious; 87 per cent. of the boys had decayed teeth.

That dental disease has increased to an alarming extent no one can deny, and there is no doubt that our artificial mode of living and our present high state of civilisation tend to produce ill-development and degeneration of the teeth.

That the primary cause of dental caries is microbic has been proved beyond a doubt. Goadby, in his work on the bacteriology of the mouth, mentions fourteen different bacteria which take an active part in the production of dental decay. Miller defines dental decay as a chemico-parasitical process consisting of two distinctly marked stages; decalcification or softening of the tissues, and dissolution of the softened residue.

Every tooth has a protective coat of enamel which completely covers its exposed surface, consisting of calcified lime salts, a small percentage of water, and practically no organic material. After a meal particles of food cling to the teeth, get lodged between proximal surfaces or in pits and grooves in the enamel; bacteria which get intimately mixed up with the food in mastication set up fermentation, and acids are formed, principally lactic acid.

Decay invariably starts in some part of the tooth where food is free to lodge, and Leon Williams has described a felt-like mass of micro-organisms on the surface of the enamel seen in microscopic section; of such parts he says: "This mass of fungus is so dense and adhesive as to make it highly improbable that the enamel is affected, except in rare and special instances, by any other acid than that which is being excreted by the bacteria at the very point where they are attached to the enamel."

Decay never starts from within, there must always be a "solution of continuity" of the enamel before dentine can be attacked by caries; a gangrenous pulp may arise from traumatism or other cause leading to abscess formation without any enamel disintegration; in this case the dead pulp becomes infected from the blood stream, but not so does caries affect the dentine.

When bacteria have succeeded in disintegrating the enamel they proceed to digest the dentine (which contains considerable organic material), and this process goes on till the pulp is laid bare; violent toothache may then result, or the pulp may slowly die and become gangrenous, with or without abscess formation. Since the primary cause of dental caries is parasitic, we may well inquire why it is so much more prevalent now than in the past.

Secondary, predisposing causes of dental disease we have already mentioned. Degeneration and a lessened resistance to all pathogenic micro-organisms is one of these. Another predisposing cause is undoubtedly heredity. Exanthemata in early childhood, as we shall show later, tend to produce flaws in the enamel predisposing to caries; other such causes are pregnancy or severe and prolonged illness, but by far the most active factor predisposing to dental caries is diet.

In the temporary dentition the greater part of that portion of the tooth which is to erupt above the gingival margin (that is to say the crown) is formed in utero; and in the permanent dentition before the age of six. After this latter age the blood stream can have very little influence on the enamel and dentine, for in dental operations it is often necessary to exterminate the pulp, and the enamel and dentine are then no more liable to caries, or to any deleterious change, than in the normal tooth. There is one exception; a vital pulp is able sometimes to form a small amount of new secondary dentine as a cap for its own protection. If then the blood stream has no influence on the structure of the tooth, it follows that diet can have no influence except an external one.

Before the age of six faulty nutrition no doubt plays an important part in adversely influencing the structure of a tooth, in the same way that faulty nutrition influences the structure of the bones in rickets, etc. We know that toxic conditions of the blood in early childhood will markedly influence tooth structure; for a child who has had one of the exanthemata will bear evidence of it in the form of pits and grooves running across the enamel, and these pits and grooves will correspond with that portion of the tooth which was being formed at the time of the exanthemata.

Diet, then, has to be considered from two aspects; the one, its influence after assimilation on the building up of tooth structure in young children; the other, its external influence on teeth generally. Röse carried out some important investigations on the influence on caries of lime in food and water. In country places poor in lime he found that 98.7 per cent. of children had dental caries, and the percentage of all diseased teeth in these children was 35.3. In places rich in lime he found that the percentage of children with caries was 79, and the percentage of all diseased teeth in these children 16.1.

One observer has stated that breast-fed children have the best teeth, and those brought up on patent foods the worst; this may be true of the temporary teeth, but it is difficult to see how it can much affect the permanent dentition, for calcification scarcely starts before the age of one year. A proper proportion of lime salts is a very necessary constituent of the diet of young children; an average wholesome diet should contain this proportion, and with this fresh air, exercise, suitable clothing, and freedom from excitement will be necessary for its proper assimilation.

The second point—the external influence of diet upon the teeth—remains to be considered. Sims Wallace states that "the cause of the prevalence of dental caries is that the natural food-stuffs are, to a large extent, ridded of their accompanying fibrous parts and prepared and consumed in a manner which renders them liable to lodge and undergo acid fermentation in the mouth, while from the same cause and the induced condition the micro-organisms of the mouth lodge and multiply and augment the rapidity and intensity of the acid fermentation."

The modern method of making bread whereby the coarser elements are extracted to render it white and soft; the habit of eating it comparatively new; highly seasoned, thoroughly cooked, softened food; and the absence from our diet of such hard teeth-cleaning things as raw apples, nuts, etc., are all conducive to dental caries. In conclusion then, we may say that dental caries is brought about by the action of bacteria, that the artificial feeding of infants produces badly formed teeth, and that the modern method of cooking and preparing food predisposes to caries by depriving the teeth and jaws of necessary activity, and by introducing into the buccal cavity a soft and sticky mass which forms a suitable nidus for bacterial activity.

